# Chronic high fat feeding restricts islet mRNA translation initiation independently of ER stress via DNA damage and p53 activation

**DOI:** 10.1038/s41598-017-03869-5

**Published:** 2017-06-19

**Authors:** Masayuki Hatanaka, Emily Anderson-Baucum, Alexander Lakhter, Tatsuyoshi Kono, Bernhard Maier, Sarah A. Tersey, Yukio Tanizawa, Carmella Evans-Molina, Raghavendra G. Mirmira, Emily K. Sims

**Affiliations:** 1 Department of Pediatrics, Indiana University School of Medicine, Indianapolis, IN USA; 2 Department of Medicine, Indiana University School of Medicine, Indianapolis, IN USA; 3 Department of Biochemistry and Molecular Biology, Indiana University School of Medicine, Indianapolis, IN USA; 4 Department of Cellular and Integrative Physiology, Indiana University School of Medicine, Indianapolis, IN USA; 5 Herman B Wells Center for Pediatric Research, Indiana University School of Medicine, Indianapolis, IN USA; 6 Center for Diabetes and Metabolic Diseases, Indiana University School of Medicine, Indianapolis, IN USA; 7 Division of Endocrinology, Metabolism, Hematological Sciences and Therapeutics, Yamaguchi University Graduate School of Medicine, Yamaguchi, Japan

## Abstract

Under conditions of high fat diet (HFD) consumption, glucose dyshomeostasis develops when β-cells are unable to adapt to peripheral insulin demands. Few studies have interrogated the molecular mechanisms of β-cell dysfunction at the level of mRNA translation under such conditions. We sought to address this issue through polyribosome profile analysis of islets from mice fed 16-weeks of 42% HFD. HFD-islet analysis revealed clear trends toward global reductions in mRNA translation with a significant reduction in the polyribosome/monoribosome ratio for *Pdx1* mRNA. Transcriptional and translational analyses revealed endoplasmic reticulum stress was not the etiology of our findings. HFD-islets demonstrated evidence of oxidative stress and DNA damage, as well as activation of p53. Experiments in MIN-6 β-cells revealed that treatment with doxorubicin to directly induce DNA damage mimicked our observed effects in islets. Islets from animals treated with pioglitazone concurrently with HFD demonstrated a reversal of effects observed from HFD alone. Finally, HFD-islets demonstrated reduced expression of multiple ribosome biogenesis genes and the key translation initiation factor eIF4E. We propose a heretofore unappreciated effect of chronic HFD on β-cells, wherein continued DNA damage owing to persistent oxidative stress results in p53 activation and a resultant inhibition of mRNA translation.

## Introduction

Type 2 Diabetes (T2D) is characterized by a combination of peripheral insulin resistance and intrinsic islet β-cell dysfunction, culminating in hyperglycemia^1^. A diet high in fat, such as the typical “Western diet,” can increase T2D risk through multiple mechanisms^2^. Chronically increased serum free fatty acids associated with obesity can increase β-cell insulin demand via reductions in insulin sensitivity, but also induce intrinsic β-cell dysfunction via endoplasmic reticulum (ER) stress, mitochondrial dysfunction, and oxidative stress^3, 4^.

To date, much of the analyses of β-cell function in the setting of obesity and insulin resistance have focused on investigation of transcriptional regulation and signal transduction pathways^5^. However, regulation at the level of mRNA translation in the β-cell has received less attention, despite a critical role for translational regulation in determining cellular protein levels as well as maintaining glucose homeostasis and β-cell function^6–10^. We have previously demonstrated that treatment of β-cells *in vitro* with the saturated free fatty acid palmitate (to mimic the lipotoxic effects of Western diets) had a dichotomous, time-dependent effect on β cell translation^11^. In the short term, palmitate treatment increased insulin production via activation of mammalian target of rapamycin complex 1 (mTORC1). By contrast, similar to previous reports of prolonged cytokine incubations, longer-term treatment with palmitate *in vitro* resulted in a translational blockade via activation of ER stress pathways and phosphorylation of eukaryotic translation initiation factor 2α (eIF2α)^8, 11^. *In vivo* studies in mice on short-term high fat diet (HFD) were consistent with activation of mTORC1 and increased mRNA translation in islets^11^. However, the effects *in vivo* of long-term exposure to HFD on islet mRNA translation and the underlying cause(s) of these effects have heretofore remained unclear.

Here, we investigated the mRNA translational effects of long-term HFD feeding through polyribosome profile (PRP) analysis of islets from C57BL6/J mice fed 16 weeks of HFD. In contrast to our work with short term HFD (using HFD with 60% of kcal derived from fat), we chose HFD chow containing 42% of kcal from fat to approximate the typical chronic human exposure to a Western diet. We hypothesized that islets from mice on chronic HFD would show decreased active β-cell translation due to activation of ER stress pathways. To our surprise, although HFD islets did display a net decrease in translational activity, this effect was not consistent with the pattern typically seen in ER stress. Based on a functional pathway analysis from a microarray performed on islets from mice treated with HFD, we identified evidence of HFD-induced activation of p53, in turn leading to reductions in β-cell translation. Our findings demonstrate that chronic HFD feeding results in a reduction in islet mRNA translation initiation that is distinct from ER stress.

## Results

### HFD feeding causes a reduction in islet mRNA translation initiation

**Figure 1:**
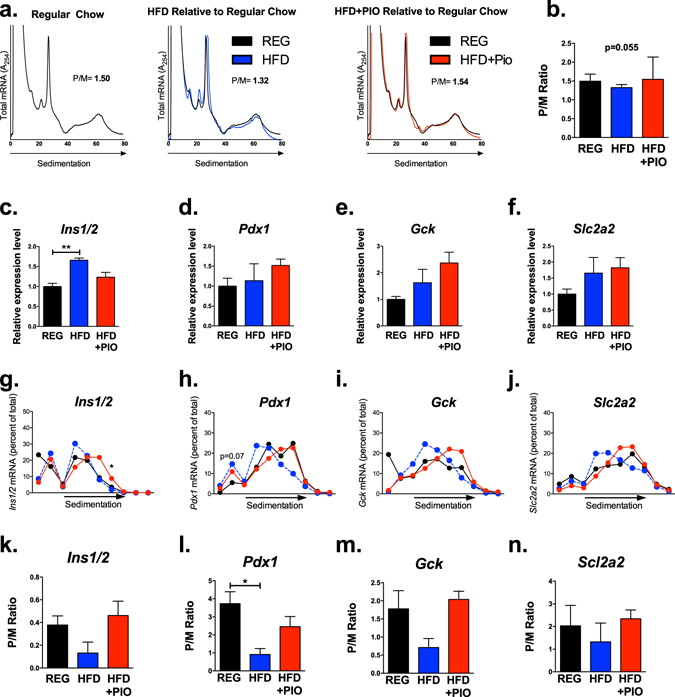
Chronic HFD induces global reductions in mRNA translation. Polyribosome profile (PRP) analysis was performed on islets from 3–5 mice on regular chow (REG), 42% high fat diet (HFD) or HFD in combination with Pioglitazone (HFD + PIO). (**a**) Representative global profiles are shown. (**b**) Polyribosome/monoribosome (P/M) ratios for each treatment group. One way analysis of variance was performed to identify between group differences. (**c**–**f**) Total input RNA expression for genes related to β cell function and identity, including *Ins1/2, Pdx1, Gck*, and *Slc2a*, was analyzed. Individual fraction transcript expression for (**g**) *Ins1/2*, (**h**) *Pdx1*, (**i**) *Gck*, and (**j**) *Slc2a*, was also analyzed. Composite curves representing average values of all animals are presented. Average values were significantly different among groups for fraction 7 of the insulin tracing and neared significance for fraction 2 of the *Pdx1* tracing. (**k**–**n**) P/M ratios were calculated for each transcript. *p < 0.05, **p < 0.01.

Effects of 16 weeks of HFD (42% kcal from fat) on metabolic characteristics of this cohort of male C57BL/6 J mice have previously been published by our group^12^. In short, chronic HFD resulted in weight gain, increased visceral fat mass and insulin resistance, increased markers of inflammation, decreased circulating adiponectin, and worsened glucose tolerance compared to mice placed on a regular chow diet (REG diet, 17% kcal from fat)^12^. To assess the effects of chronic HFD on islet mRNA translation, we performed PRP analysis on islets isolated from mice in each diet group. PRP analysis involves isolating total cellular RNA, followed by use of a sucrose gradient to separate RNA based on the nature and number of associated ribosome units^9, 13^. The representative PRPs from mice on REG diet or HFD in Fig. 1a show the association of RNAs with the 40S and 60S ribosomal subunits and monoribosome (80S), which collectively indicate initiating or inactively translating RNA species. Conversely, association of RNA species with polyribosomes (more than two monoribosomes) generally indicates active translation. The HFD PRP shown in Fig. 1a demonstrates the decreased presence of mRNA transcripts in the polyribosome-associated portion of the curve, suggesting a net reduction in the rate of mRNA translation initiation (with subsequent “run-off” of polyribosomes). Correspondingly, average polyribosome/monoribosome (P/M) ratios in islets of animals on HFD tended to be reduced compared to mice on REG diet (Fig. 1b). By contrast, islets from mice on a C57BLKS/J background, which compensated for HFD treatment with a reduction in food intake, demonstrated no differences in global PRPs in response to HFD (Supplemental Fig. 1)^12^.

Because subtle shifts in global PRPs can reflect large effects on ribosome engagement of specific subsets of abundant mRNAs, we evaluated the ribosome engagement of mRNAs encoding insulin (*Ins1/2*), Pdx1 (*Pdx1*), glucokinase (*Gck*), and Glut2 (*Slc2a2*), all known to be important for β-cell function and identity. As shown in Fig. 1c–f, the total levels of each of these mRNAs were either unchanged, or in the case of *Ins1/2*, increased in islets from mice fed HFD (compared to REG diet). However, as shown in Fig. 1g–j, HFD feeding consistently led to a shift of transcripts towards monosome-associated fractions, suggesting decreased translation of multiple crucial β-cell mRNAs. Quantification of P/M ratios for individual mRNAs revealed a significant reduction in P/M ratio for *Pdx1* transcripts in islets from mice on HFD (Fig. 1l).

### The reduction in translation initiation with HFD feeding is not secondary to ER stress

**Figure 2:**
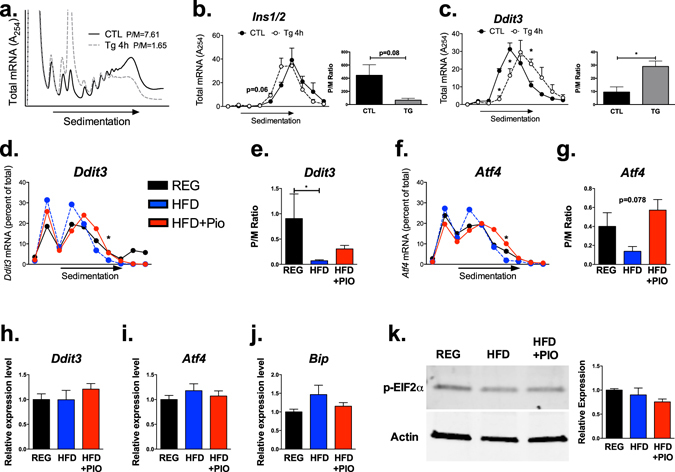
HFD-induced reductions in mRNA translation are not caused by ER Stress. (**a**–**c**) MIN-6 β cells were treated with thapsigargin (Tg) for 4 hours as a positive control for ER stress induction (n = 3 per group). (**a**) Representative global PRPs. For each fraction, transcript expression for (**b**) *Ins1/2* and (**c**) *Ddit3* were analyzed. (**d**) Composite curves representing average *Ddit3* transcript expression in PRP fractions from islets of mice treated with regular chow (REG), 42% high fat diet (HFD) or HFD in combination with Pioglitazone (HFD + PIO). (**e**) P/M ratios for *Ddit3* transcripts in each diet treatment group. (**f**) Average *ATF4* transcript expression in PRP fractions from islets of mice in each diet group. (**g**) P/M ratios for *ATF4* transcripts in each diet treatment group. (**h**–**j**) qRT-PCR analysis of total RNA inputs for genes associated with ER stress signaling, including *Ddit3, Atf4, and Bip*.(**k**) Representative immunoblot and quantification of phosphorylated eukaryotic translation initiation factor 2α (p-eIF2α) expression among diet groups. n = 3–5; *p < 0.05.

Next, we endeavored to understand if the observed reduction in translation initiation of key β-cell genes was secondary to ER stress induced by chronic HFD. As a positive control for robust ER stress, MIN6 β-cells were treated with thapsigargin, an inhibitor of the sarco/endoplasmic reticulum Ca2 + ATPase. As shown by the representative PRPs in Fig. 2a, thapsigargin induced a translation initiation block, with reduced P/M ratios compared to control cells (similar to that observed in islets of HFD fed mice). qRT-PCR performed on individual fractions from the PRPs revealed a shift in *Ins1/2* transcripts from polyribosome-associated fractions to monosome-associated fractions (Fig. 2b). However, consistent with known effects of ER stress and the unfolded protein response, wherein several “privileged” mRNAs escape the general suppression of translation initiation, the mRNA encoding C/EBP homologous protein (Chop) (*DNA Damage Inducible Transcript 3, Ddit3*) demonstrated a shift toward polyribosome engagement with thapsigargin treatment, suggestive of increased translation (Fig. 2c)^8, 9^. In direct contrast to these findings, analysis of islets from mice on chronic HFD failed to demonstrate privileged translation of *Ddit3* or *Activating transcription factor 4* (*Atf4*) mRNAs (Fig. 2d–g). Instead, transcripts for both *Ddit3* and *Atf4* were shifted toward the monoribosome-associated fractions, with a significant reduction in the P/M ratio for *Ddit3* in islets from mice treated with HFD. Along these lines, no significant differences between treatment groups were found in transcripts isolated from total RNA for multiple genes associated with ER stress signaling (Fig. 2h–j). Next, we performed immunoblots to assess for differences in islet phosphorylated eIF2α (p-eIF2α), and were unable to detect differences among treatment groups. Although these findings do not preclude activation of other ER stress pathways, in aggregate, our data suggest that negative effects of chronic HFD on islet translation were not consistent with ER Stress-induced repression of translation initiation.

**Figure 3:**
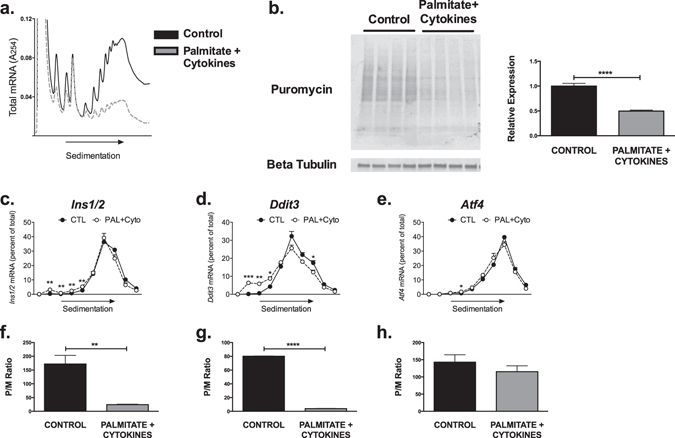
Modeling chronic β cell lipotoxicity to mimic *in vivo* effects of chronic HFD. MIN-6 β cells were treated with 40 hours of palmitate + a cytokine mix to mimic chronic lipotoxicity and inflammation (n = 4). (**a**) Representative global PRP tracing. (**b**) Puromycin incorporation of cells from both treatment groups, with quantification of total band intensities. (**c**–**e**) PRP analysis of individual transcripts for *Ins1/2, Ddit3*, and *Atf4* by fraction.(**f**–**h**) Quantification of P/M Ratios for each transcript. *p < 0.05, **p < 0.01, ***p < 0.001, ****p < 0.0001.

Next, to better model the effects of chronic HFD on β-cell translation, we treated MIN6 β-cells with a mixture of palmitate and proinflammatory cytokines (to mimic the chronic lipotoxicity and inflammation expected in response to HFD feeding) for 40 hours. PRP analysis revealed a significant reduction in polyribosome-associated RNAs, consistent with a net decrease in translation initiation (Fig. 3a). The reduction in polyribosome-associated RNAs correlated with a decrease in protein synthesis, as puromycin incorporation was decreased in cells treated with palmitate and cytokines (Fig. 3b). However, in contrast to analyses presented in Fig. 2, and consistent with our results in islets from HFD treated mice, evaluation of individual mRNAs demonstrated a monoribosome shift of *Ins1/2*, *Ddit3*, and *Atf4* messages (Fig. 3c–e; P/M ratios quantified in Fig. 3f–h).

### HFD feeding causes DNA damage, oxidative stress, and activation of p53 signaling

**Figure 4:**
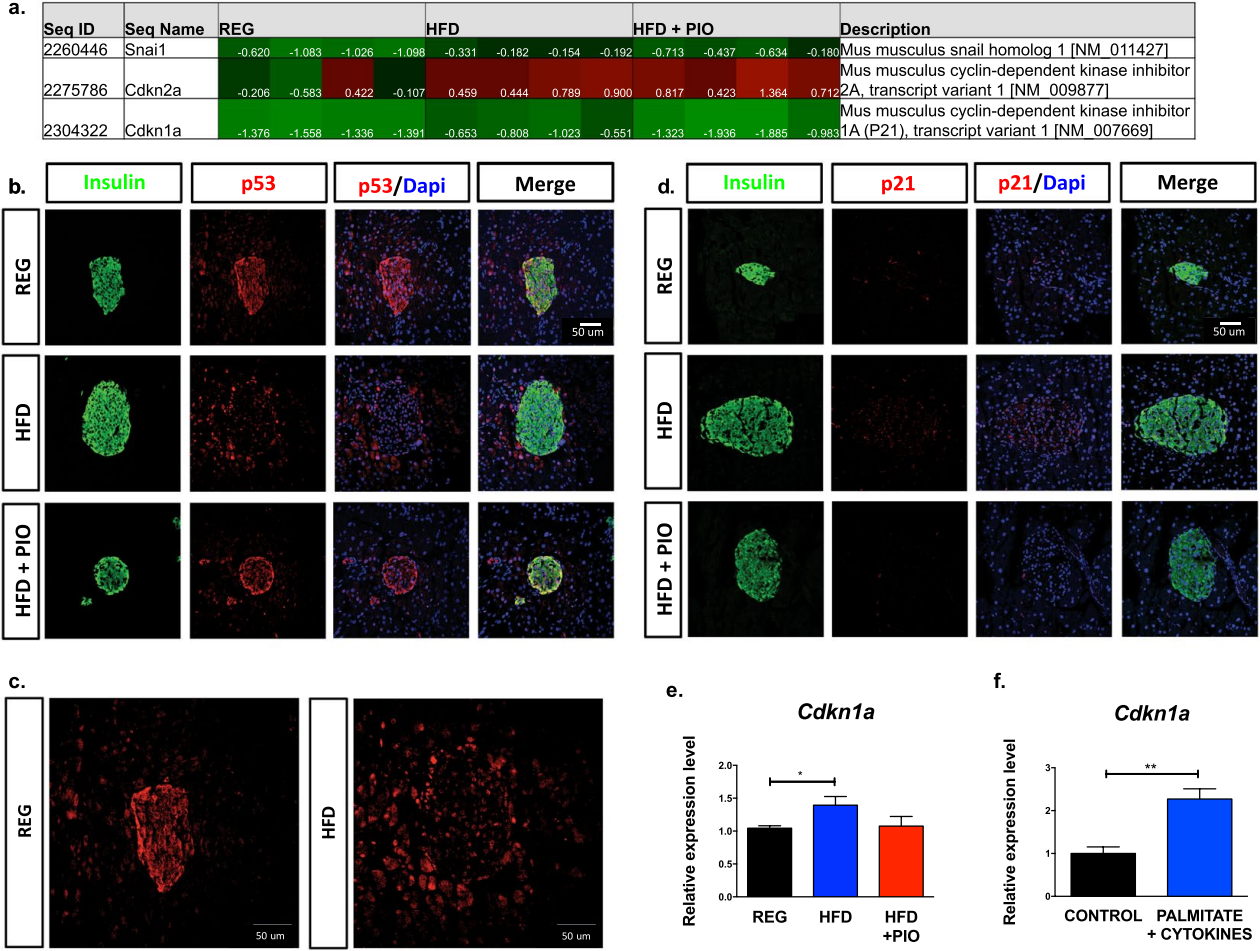
Evidence of p53 activation in islets from HFD treated mice. (**a**) Microarray functional grouping analysis of islets from each diet group. (n = 4) (**b**) Representative immunofluorescent staining of p53, insulin, and DAPI for islets from each treatment group. (n = 3) (**c**) Enlarged image of p53 staining in REG and HFD treatment groups. (**d**) Representative immunofluorescent staining of p21, insulin, and DAPI for islets from each treatment group. (n = 3) (**e**) qRT-PCR analysis of total islet RNA for *Cdkn1a* expression. (n = 4) (**f**) qRT-PCR analysis for *Cdkn1a* expression in MIN-6 β cells treated with 40 hours of palmitate + a cytokine mix to mimic chronic lipotoxicity. *p < 0.05, **p < 0.01.

To identify a molecular etiology for our results, we performed a functional grouping analysis of our previous published islet microarray data from these dietary cohorts^12^. Analysis revealed an upregulation of p53 signaling in islets from mice on chronic HFD. A heat map of individual genes identified is displayed in Fig. 4a. To verify this upregulation, we performed immunofluorescence staining of pancreas tissue for p53. As shown in Fig. 4b,c, p53 showed increased nuclear staining in islets from mice fed a HFD compared to REG diet, a result consistent with p53 activation. Along these lines, immunostaining for the established p53 target gene *Cyclin dependent kinase inhibitor 1a* (*Cdkn1a*), encoding p21, revealed an upregulation in islets from mice fed a HFD (Fig. 4d)^14^. qRT-PCR from islets from animals in both treatment groups confirmed a significant upregulation in *Cdkn1a* transcripts in islets from HFD-fed mice (Fig. 4e). qRT-PCR was also performed using our *in vitro* model of cells treated with palmitate and proinflammatory cytokines to mimic chronic HFD. A significant increase in *Cdkn1a* expression was also present in cells treated with palmitate and cytokines, confirming that the translation initiation block observed in our *in vitro* model was similarly associated with p53 activation (Fig. 4f). Because our previously published data did not demonstrate any evidence of β-cell apoptosis in C57BL/6 J mice on HFD, we also reviewed our array data for relative expression of genes that would be expected to be increased by activation of a p53-induced apoptotic program^12, 15^. As shown in Supplemental Figure 2 and consistent with our previous data, relative expression of these genes was not impacted by HFD.

**Figure 5:**
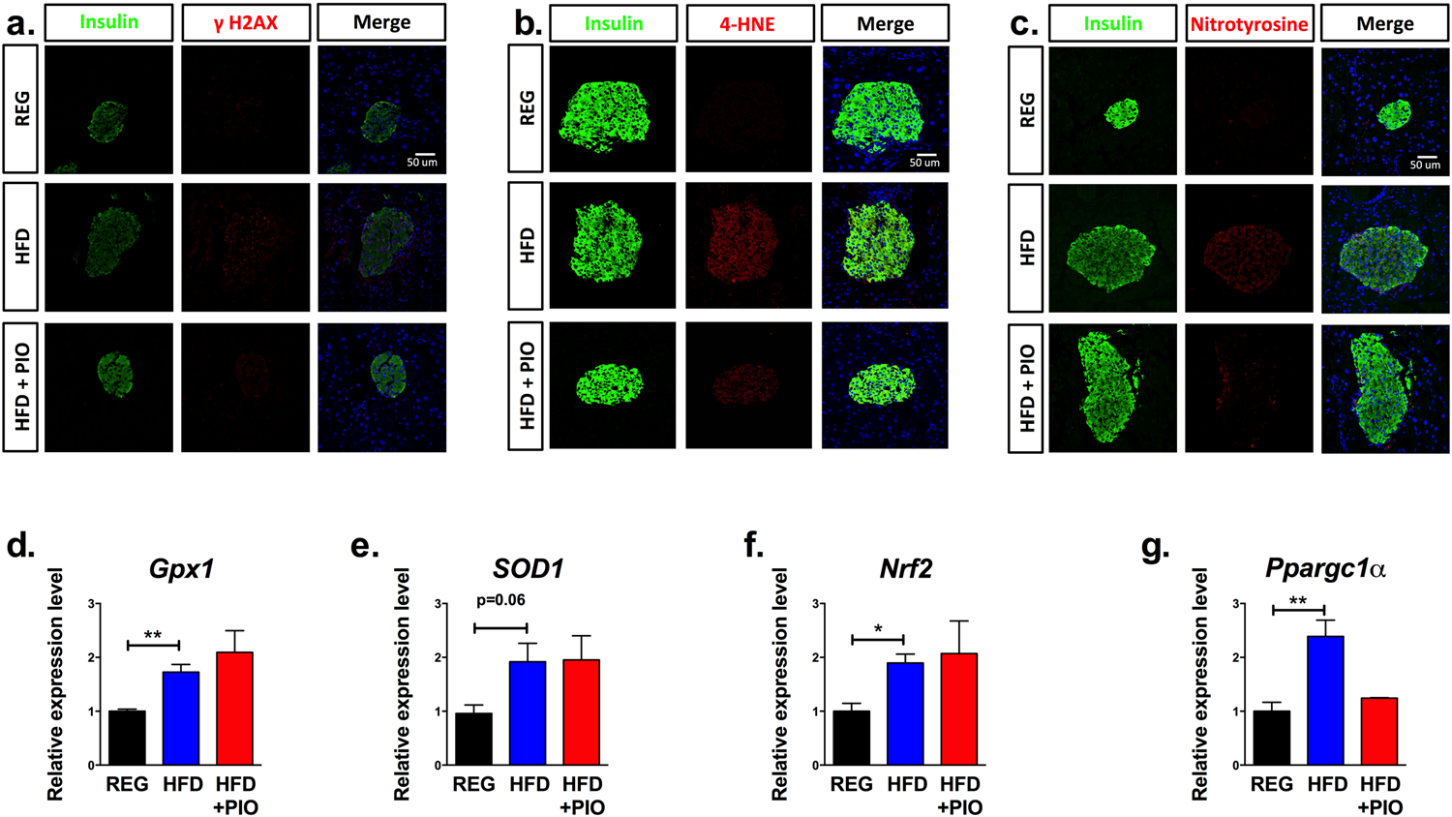
Evidence of DNA damage and oxidative stress in islets from HFD treated mice. (**a**) Representative immunofluorescent staining of γH2AX, insulin, and DAPI for islets from each treatment group. (**b**) Representative immunofluorescent staining of 4-hydroxynonenal (4-HNE), insulin, and DAPI for islets from each treatment group. (**c**) Representative immunofluorescent staining of nitrotyrosine, insulin, and DAPI for islets from each treatment group. (**d**–**g**) qRT-PCR analysis of total islet RNA for antioxidant genes, including *Gpx1, SOD1, Nrf2*, and *Ppargc1α*. (n = 3) *p < 0.05, **p < 0.01.

Activation of p53 is associated with DNA damage^16^. To identify if the upregulation in p53 signaling occurred in association with islet DNA damage, staining for γH2AX, a phosphorylated histone marking double-stranded DNA breaks, was performed^17^. As shown in Fig. 5a, increased γH2AX staining was observed in islets from HFD-fed mice, but not in islets of REG diet-fed animals. We suspected that chronic increases in free fatty acids and systemic inflammation induced by HFD may have led to increased islet reactive oxygen and nitrogen species and oxidative stress, which could explain the observed increase in DNA damage. Staining for 4-hydroxynonenal (4-HNE) and nitrotyrosine verified increased oxidative stress in HFD islets (Fig. 5b,c). Lastly, to identify activation of antioxidant genes as an alternative measure of reactive oxygen species production, qRT-PCR of islets from each dietary group revealed a significant upregulation of *Gpx1* (encoding Glutathione peroxidase 1), *Nfe2l2* (encoding Nuclear factor E2 Related 2, or Nrf2), and *Ppargc1a* (encoding peroxisome proliferator-activated receptor gamma coactivator 1α) in HFD-treated animals, consistent with a response to oxidative stress (Fig. 5d–g).

**Figure 6:**
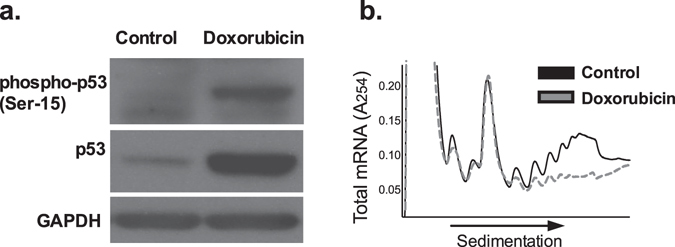
β cell DNA damage to induce p53 activation leads to global reductions in mRNA translation. MIN-6 cells were treated with doxorubicin to directly induce DNA damage. (**a**) Protein levels of both total and phospho-p53 were assayed after 12 hours of treatment with doxorubicin. (**b**) Global PRP analysis after 48 hours of doxorubicin treatment.

To establish a direct causal link between p53 activation and inhibition of translation initiation, MIN6 β-cells were treated with doxorubicin to induce DNA damage and activate p53. Immunoblot analysis performed on cells after 12 hours of treatment demonstrated increased levels of phopho-p53 (Ser15), confirming p53 activation (Fig. 6a). After 48 hrs of treatment, PRP analysis demonstrated a reduction in the polyribosome-associated RNAs, consistent with a net reduction in translation initiation (Fig. 6b).

### PPAR-γ activation reverses the translation inhibitory effect of HFD feeding

We wanted to test whether our findings of a HFD-induced translational initiation block could be reversed by reduction of oxidative stress. To this end, as PPAR-γ agonists are known to reduce β-cell oxidative stress, we evaluated mice that received HFD compounded with pioglitazone (HFD + PIO)^18^. Strikingly, PIO treatment reversed the reduction in islet translation initiation seen with chronic HFD feeding. This effect was observed in both the PRP analysis (Fig. 1b) and for individual mRNAs evaluated, with a shift in mRNAs towards the polyribosome-associated portions of the curve, indistinguishable from animals on REG diet (Figs 1g–n and 2d–g). Islet γH2AX, 4-HNE, and nitrotyrosine staining in HFD + PIO fed animals were more comparable to animals on REG diet (Fig. 5a–c). Correspondingly, islets from mice on HFD + PIO also had a reversal in p53 signaling on microarray analysis (Fig. 4a), reduced nuclear p53 compared to HFD islets (Fig. 4b,c), and p21 levels indistinguishable from mice on REG diet (Fig. 4d,e).

### Potential Etiologies of p53-related Translation Initiation Blockade

**Figure 7:**
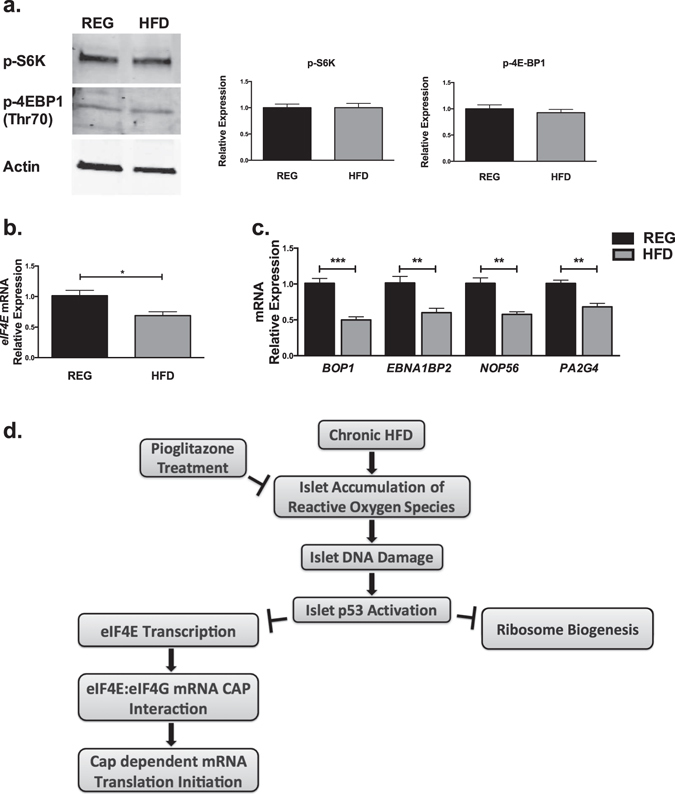
Mechanisms of HFD effects on β cell mRNA translation. (**a**) Immunoblots for phosphorylated S6 kinase at T389 (p-S6K) and phosphorylated eIF4E binding protein 1 at T70 (p-4E-BP1) in islets from mice on regular diet and HFD. (**b**) q-PCR evaluating relative expression of *eIF4E* transcripts. (**c**) q-PCR evaluating relative expression of ribosome biogenesis genes *BOP1, EBNA1BP* *, NOP56*, and *PA2G4*. n = 3–4; *p < 0.05, **p < 0.01, ***p < 0.001. (**d**) Proposed mechanisms of HFD-induced reductions in β cell translation.

Next we investigated downstream targets of p53 activation that could contribute to our observed mRNA translational phenotype. In our previous work, we demonstrated that mTORC1 played an important role in regulation of islet translation in response to short term, 60% HFD^11^. p53 signaling has been demonstrated to directly activate Sestrin genes to inhibit mTORC1 activity^19^. Therefore, we performed immunoblots for two major translation promoting targets of mTORC1, phosphorylated S6 kinase at T389 (p-S6K) and phosphorylated eIF4E binding protein 1 at T70 (p-4E-BP1), to evaluate the possibility of reductions in mTORC1 activity in islets from mice on HFD (Fig. 7a)^19^. However, no differences in either p-S6K or p-4E-BP1 were detected. Next, because p53-related inhibition of c-myc activity can lead to reductions in the transcription of the important eukaryotic initiation factor eIF4E, we performed qRT-PCR for *eIF4E transcripts* (Fig. 7b)^20^. Here, we observed a significant reduction in expression of *eIF4E* transcripts in islets from mice on HFD. Lastly, as p53 activation has been associated with reductions in genes important for ribosome biogenesis, we quantified relative expression of a panel of ribosome biogenesis genes previously shown to be downregulated by p53 induction (*BOP1, EBNA1BP2, NOP56*, and *PA2G4;* Fig. 7c)^21^. Relative expression of each of these ribosome biogenesis mRNAs was decreased in islets from mice on HFD.

## Discussion

The impact of HFD on islet β-cell function is complex, with effects that occur both directly and indirectly (via insulin resistance and systemic inflammation). In the short term, elevations in free fatty acids as a result of HFD lead to activation of mTORC1, glycerolipid-free fatty acid cycling, and compensatory replication in the β-cell, giving rise to enhanced insulin release and increased β-cell mass^11, 22–24^. Chronic exposure to HFD and elevations in free fatty acids are known to have direct effects on β-cell function and survival via islet inflammation, activation of oxidative and ER stress pathways, and alterations in transcription factor localization and expression^4, 25–27^. However, the effects *in vivo* of chronic HFD on β-cell mRNA translation have been poorly characterized. In this study, we identified a novel relationship between HFD-induced oxidative stress leading to islet DNA damage and activation of p53 signaling, and subsequent translational inhibition of key genes relevant to β-cell function (Fig. 7). Importantly, our findings were distinct from previously reported effects of prolonged treatment with cytokines alone, which induced a classic phenotype of ER stress-induced translation initiation blockade, with increased phosphorylation of eukaryotic translation initiation factor 2α (p-eIF2α) and preferentially increased translation of mRNAs encoding Chop and ATF4, as well as preservation of *Pdx1* mRNA translation^8, 28^.

The β-cell is known to be sensitive to excessive ROS accumulation and oxidative stress^29^. Accordingly, oxidative stress has been proposed as an important contributor to β-cell lipotoxicity, via increased fatty acid β oxidation or NADPH oxidase activity, leading to generation of pathologic levels of reactive oxygen species and subsequent β-cell dysfunction and death^3, 30, 31^. Oxidative stress is an established cause of DNA damage, including double-strand DNA breaks, and importantly, both DNA damage and oxidative stress markers are increased in islets from T2D patients^32, 33^. These effects can be mitigated by treatment with antioxidants, or therapies like PPAR-γ agonists, which induce antioxidant expression and reduce accumulation of the reactive oxygen and nitrogen species associated with oxidative stress^3, 5, 18, 30^.

p53, a classic tumor suppressor, is a crucial component of the physiologic response to DNA damage, such as that induced by oxidative stress^16^. Activation of p53 and its downstream mediators, such as p21^CIP1/WAF1^, can result in temporary cell cycle arrest, allowing for activity of DNA repair programs, or alternatively, can result in tissue dysfunction, senescence, or apoptosis^16^. Our data suggest that in the context of chronic HFD, p53 contributes primarily to islet senescence, rather than activation of apoptotic signaling. Although p53 was first recognized as a transcription factor, mounting evidence points to p53 as an important regulator of translation^34^. Control of translation can occur through p53-mediated regulation of specific miRNAs, long noncoding RNAs, and RNA binding proteins that control translation of specific mRNAs, or via more global effects^34, 35^. Experiments employing simultaneous RNA sequencing of global transcriptomes and polyribosome-associated mRNAs to determine effects on transcription and translation have revealed that p53 activation modulates translation but not transcription of a large subset of mRNAs, including ribosomal proteins and translation factors^21, 35^.

Several mechanisms underlying the effect of p53 on global protein synthesis have been identified. CAP-dependent initiation of translation is normally regulated by activation of mitogenic pathways, such as mammalian target of rapamycin (mTOR)^34^. Through direct activation of mTOR inhibitors, p53 is able to inhibit mTOR signaling resulting in global repression of translation^21^. p53 binding and inhibition of c-myc results in decreased c-myc stimulated transcription of eukaryotic initiation factor eIF4E, which directly recognizes and binds the 5′ mRNA CAP^20^. p53 activation also interferes with the interaction of eIF4E and eIF4G, ultimately reducing RNA translation initiation^36, 37^. p53 also affects global translation patterns via inhibition of ribosome biogenesis. Transcriptome analysis revealed that p53 activation repressed numerous genes coding for ribosomal proteins or involved in regulation of ribosome biogenesis^21^. In addition, p53 is able to inhibit the recruitment/activity of both RNA Polymerase I and III at the rDNA promoters^38, 39^. Alternatively, p53 has also been identified to target genes regulating chemical modifications of ribosomal RNAs, which could affect their functions^34^. Our data point to reductions in islet transcription of eIF4E as well as several genes involved in ribosome biogenesis in association with activation of p53 signaling. Future work will further elucidate these relationships, as well as other downstream effectors of p53 activation within the islet.

Several other reports have explored the role of p53 in the β-cell. Increased pancreatic radiation doses incurred for cancer treatment in childhood are associated with increased development of T2D in adulthood, suggesting a potential effect of radiation-induced DNA damage^40^. Intriguingly, evidence of DNA damage and p53 activity, including increased p21 expression, has previously been demonstrated in islets from *db*/*db* mice and humans with T2D^41–43^. Mice overexpressing Δ40p53, an isoform of p53 that stabilizes the full length protein, demonstrated increased p21 expression, reduced cyclin D2 and Pdx-1 expression, and reduced β-cell proliferation and β-cell mass, eventually leading to hypoinsulinism and frank diabetes^44^. Genetic knockout of β-cell p53 was able to prevent oxidative stress-induced β-cell apoptosis in a mouse model of congenital hyperinsulinism^41^. Interestingly, genome-wide association studies have identified an association between a polymorphism in p53 codon 72 and T2D susceptibility^45, 46^.

Because of technical limitations associated with analyses of large numbers of mice, our data were obtained from several time-points occurring over a 4-week period (12 weeks, 14 weeks, and 16 weeks), with global effects on PRPs observed after 16 weeks of diet. Not all studies were able to be performed at every time-point, and effects of chronic HFD and ROS accumulation may have continued to evolve over this time period. However, we observed evidence of p53 activation at each time-point, suggesting that activation of this signaling pathway plays an important role in the islet response to HFD that persists over time. Along these lines, our observation that the translation initiation blockade was inconsistent with the classic translation blockade associated with ER stress may be a reflection of an evolving chronic response to activation of these pathways.

HFD feeding in our study resulted in evidence of a blockade in translation initiation, with subtle shifts towards the monoribosome-associated portion of the PRP curve for most transcripts evaluated. These findings are in line with other mild β-cell effects found in this cohort, including the absence of defects in GSIS or of β-cell death^12^. In part, these findings likely reflect our choice of a more moderate high fat diet (42% of kcal from fat), in contrast to other studies utilizing diets with 60% of kcal from fat, in which abnormalities in GSIS were identified^47, 48^. Our own previously reported observations of short-term effects of HFD were performed using a 60% HFD^11^. It is possible that we may have observed more pronounced or different effects with a higher percentage HFD. However, our decision to treat with Western diet in this work was made in an attempt to mimic chronic HFD and obesity encountered by humans, with resulting findings physiologically relevant to human diabetes. Furthermore, subtle shifts in the global PRP reflect substantial changes in the translation of numerous individual transcripts, as we observed by demonstrating similar patterns in translation of multiple β-cell function and ER stress transcripts. Certainly, even small changes in translation of key β-cell function and survival genes, over years of accumulated DNA damage, could collectively lead to larger scale effects on β-cell function. Taken together, our data provide evidence that chronic HFD feeding results in a translational block in islets that is referable to activation of p53 and not an induction in classical ER stress.

## Methods

### Animals, Islets, and Cell Lines

As previously described, male C57BL/6 J and C57BLKS/J mice were obtained from Jackson Laboratories (Bar Harbor, ME), and maintained under protocols approved by the Indiana University School of Medicine Institutional Animal Care and Use Committee in accordance with the Association for Assessment and Accreditation of Laboratory Animal Care guidelines. Beginning at 8 weeks of age, for 12–16 weeks, animals were fed either regular chow containing 17% calories from fat (REG diet), high fat diet containing 42% calories from fat (HFD), or high fat diet compounded by Harlan-Teklad Global with 140 mg/kg of pioglitazone (HFD + PIO), calculated to provide a dose of 20 mg/kg/day of pioglitazone^12^.

Mouse islets were isolated from collagenase-perfused pancreata and cultured in RPMI 1640 medium and allowed to recover overnight prior to experimentation as previously described^49^. For PRP experiments, islets were transferred into cold RPMI medium containing 5 mM glucose after isolation and used immediately. The MIN6 mouse insulinoma cell line was maintained in culture conditions as described^11^. MIN6 cells were treated with 1 μM thapsigargin, 1 μM doxorubicin, or 0.5 mM palmitate (Sigma-Aldrich) in combination with a cytokine mix of 5 ng/ml IL-1β, 10 ng/ml TNF-α, and 100 ng/ml IFN-γ for indicated time periods. For all cell line data, results represent the means of at least three independent experiments.

### Polyribosome Profile (PRP) Experiments

PRP experiments with MIN6 cells and islets were performed as previously described^8^. Briefly, a portion of the cell lysate was preserved as the input sample to determine total mRNA levels. Lysates were passed through a 10–50% sucrose gradient and fractionated using a BioComp piston gradient fractionator. RNA absorbance at 254 nm was recorded using an in-line UV monitor and fractions were collected. Monoribosome-associated RNA was collected from fractions 1 to 5 and polyribosome-associated RNA was collected from fractions 6–10. Global polyribosome-to-monoribosome (P/M) ratios were quantitated by calculating the area under the curve corresponding to the polyribosome peaks (more than two ribosomes) divided by the area under the curve for the monoribosome (80S) peak. Total RNA from each fraction was reverse transcribed and subjected to qRT-PCR. P/M Ratios for each mRNA were quantitated by calculating the area under the curve for fractions 7–9, divided by the area under the curve for fractions 2–3. PRP studies on islets were performed after 16 weeks of treatment.

### Immunoblot analysis

Whole-cell extracts from cell lines and islet lysates were prepared and subjected to immunoblot analysis as described previously^11^. For immunoblotting of puromycin incorporation into protein, puromycin was added to the culture medium at 1 ug/mL during the final 15 minutes of incubation. Cells were then washed twice with cold PBS and lysed. Immunoblot analyses were performed after separation of protein extracts on a 4% to 20% gradient SDS-polyacrylamide gel and were visualized using fluorescently labeled primary antibody to puromycin (mouse anti-puromycin, 1:1000 concentration, Kerafast, Catalog number EQ0001), p-4EBP1 (rabbit anti-p-4EBP1, 1:1000, Cell Signaling Technology, number 9455), p-p70 S6K (Thr389) (rabbit anti-p-p70S6K, 1:1000, Cell Signaling Technology, number 2708), p-eIF2α (rabbit anti-p-eIF2 α, 1:1000, Cell Signaling Technology, number 9721), and secondary antibodies (Li-Cor Biosciences) and quantified using a Li-Cor Odyssey scanner and Image J 1.38x^50^.

### Quantitative real-time RT-PCR (qRT-PCR)

Total RNA from MIN6 cells and islets was recovered using an RNeasy kit (Qiagen), reverse transcribed, and subjected to qRT-PCR using SyBR Green based methodology^11^. Data for input RNA were normalized to *TATA box binding protein* (*Tbp*) or *Glyceraldehyde-3-Phosphate Dehydrogenase* (*GAPDH*) message. Primer sequences were previously described for *Insulin*, *Pdx1, Gck, Slc2a2, Ddit3*, *Atf4*, *Tbp*, *Gpx1*, *Nrf2*, and *Ppargc1a* ^8, 51–54^. Other primer sequences used were as follows: *Block of proliferation 1* (*BOP1*)*:* forward: 5′-GGCCCAACATGAATATGAAG-3′, reverse: 5′-TTGTAGATACGTTTGCCATC-3′; *EBNA1 Binding Protein 2* (*EBNA1BP2*)*:* forward: 5′-ATAAGCTGGATTTCCTGGAG-3′, reverse: 5′-ATTAGGCCCTTTACTCATCTG-3′; *NOP56*: forward: 5′-CCAGAGGAGTGTGAGGAGGTA-3′, reverse: 5′-GAGACAGGTGGGTCTTCCATTCC-3′; *Proliferation-Associated 2G4 PA2G4*: forward: 5′-GAAGGAGGGTGAATTTGTTG-3′, reverse: 5′-ATCTTGAACCTCCATCTCAG-3′. For *Cdkn1a* and *gapdh*, Taqman gene expression master mix reagents and gene expression assay probes were used (Applied Biosystems). Islets used for both immunoblot and PCR were isolated after 16 weeks of diet.

### Microarray analysis

After 12 weeks of diet, Agilent Whole Mouse Genome Oligo Microarray was performed on islets from 4 mice per treatment group by MACs Molecular; description and full results have previously been reported^12^. Functional grouping and annotation analysis of these results were performed by Miltenyi Bioinformatics.

### Immunofluorescence

After 14 weeks of diet, pancreata from 3 mice per treatment group were fixed by cardiac perfusion with 4% paraformaldehyde, paraffin embedded, and sectioned longitudinally at 5 μm intervals. Immunofluorescence experiments were performed on 2 sections per animal for insulin (guinea pig anti-porcine, Abcam, 1:250, AB7842), 4-HNE (rabbit anti-4HNE, Abcam 1:1000, AB66155), nitrotyrosine (rabbit anti-mouse, Millipore, 1:250, AB5411), p21 (rabbit anti-mouse, Santa Cruz, 1:250, sc397), p53 (rabbit anti-mouse, Santa Cruz, 1:250, sc6243), γH2AX pSer140 (mouse anti-mouse; Novus Biologicals, 1:150, NB100-74435), and nuclei (diamidino-2-phylindole- DAPI). Secondary antibodies were goat anti-rabbit antibody conjugated to Alexa Fluor 488 (Molecular Probes, 1:500) and goat anti-guinea pig antibody conjugated to Alexa Fluor 555 (Molecular Probes, 1:500). An SM 700 (Zeiss, Thornwood, NY) confocal equipped with an Orca ER charge-coupled device camera (Hamamatsu Photonics, Hamamatsu City, Japan) was used to acquire digital images.

### Statistics

Statistical analyses were performed using GraphPad Prism (GraphPad Software, La Jolla, California). Student’s t-tests were used for comparison between treatment and control groups. One-way ANOVA with Dunnet’s post-test, or for nonparametric distributions, Kruskal-Wallis test with Dunn’s test for multiple comparisons, were utilized when comparing >2 groups. For all analyses, a p value of ≤0.05 was considered significant.

### Data availability statement

The datasets generated during the current study are available from the corresponding author on reasonable request. The dataset from the microarray analyzed in the current study has been deposited in NCBI’s Gene Expression Omnibus and is accessible through GEO Series accession no. GSE51055 (http://www.ncbi.nlm.nih.gov/geo/query/acc.cgi? acc=GSE51055).

## Electronic supplementary material

Supplemental Figures
