# Supplementary material for: Chronic high fat feeding restricts islet mRNA translation initiation independently of ER stress via DNA damage and p53 activation
Source: Sci Rep. 2017 Jun 19;7:3758. doi: 10.1038/s41598-017-03869-5 (PMC5476640; doi:10.1038/s41598-017-03869-5)
Supplement: Supplementary file 1 — Supplemental Figures [file 41598_2017_3869_MOESM1_ESM.pdf]

Chronic high fat feeding restricts islet mRNA translation initiation independently of ER stress via DNA damage and p53 activation

Masayuki Hatanaka[1,5,6,7], Emily Anderson-Baucum[2,6], Alexander Lakhter[1,5,6], Tatsuyoshi Kono[2,6], Bernhard Maier[1,6], Sarah A. Tersey[1,6], Yukio Tanizawa[7], Carmella Evans-Molina[2,3,4,5,6], Raghavendra G. Mirmira[1-6], and Emily K. Sims*[1,5,6]

[1]Departments of Pediatrics, [2]Medicine, [3]Biochemistry and Molecular Biology, [4]Cellular and Integrative Physiology, the [5]Herman B Wells Center for Pediatric Research, and the [6]Center for Diabetes and Metabolic Diseases, Indiana University School of Medicine, Indianapolis, IN, USA
[7] Division of Endocrinology, Metabolism, Hematological Sciences and Therapeutics, Yamaguchi University Graduate School of Medicine, Yamaguchi, Japan

***To whom correspondence should be addressed:** Emily K. Sims, Indiana University School of Medicine, 635 Barnhill Drive, MS2031, Indianapolis, IN 46202, USA; Tel: (317) 944-3889; Fax (317) 944-3882. Email: eksims@iu.edu.

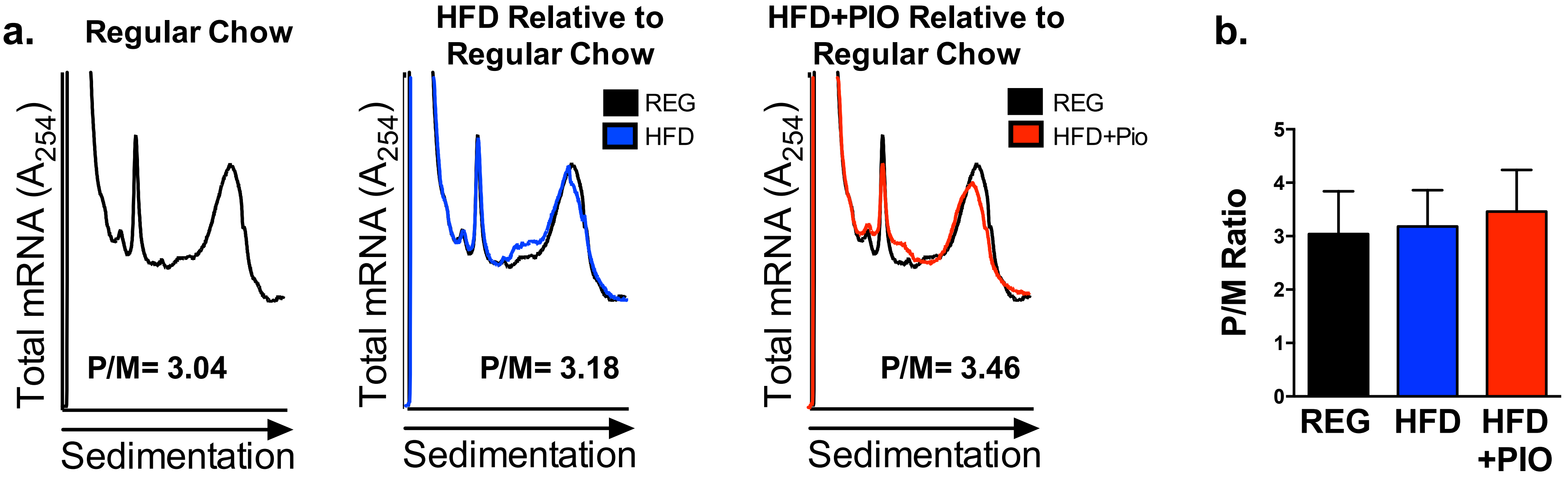

Supplemental Figure 1. HFD does not induce changes in global islet translation initiation in mice on a C57BLKS/J background. PRP analysis was performed on islets from C57BLKS/J mice that had been treated with HFD for a 16 week period. **a.** Representative global profiles for different treatment groups. **b.** Quantification of P/M ratios for each group.

Seq ID	Seq Name	REG				HFD				HFD + PIO				Description
2257190	Bax	0.116	0.087	0.217	0.023	0.056	0.121	0.130	0.338	0.253	0.159	0.316	0.243	Mus musculus BCL2-associated X protein (Bax), [NM_007527]
2269283	Bbc3	-0.431	-0.286	0.020	-0.356	-0.235	-0.462	-0.388	-0.021	-0.045	-0.217	-0.595	-0.529	Mus musculus BCL2 binding component 3 (Bbc3) [NM_133234]
2275795	Noxa1	0.416	0.209	0.360	-0.266	1.628	0.039	0.196	-0.168	0.503	0.043	0.758	-0.039	Mus musculus NADPH oxidase activator 1 (Noxa1), transcript variant 1 [NM_172204]
2294288	Perp	-1.139	-0.675	-1.010	-0.926	-1.085	-1.052	-0.794	-1.352	-1.094	-0.679	-1.091	-0.732	Mus musculus PERP, TP53 apoptosis effector (Perp) [NM_022032]
2305626	Bid	-0.319	0.033	-0.054	-0.090	-0.222	-0.227	-0.026	0.381	-0.263	0.173	1.344	-0.343	Mus musculus BH3 interacting domain death agonist (Bid) [NM_007544]

Supplemental Figure 2. HFD does not induce activation of p53 apoptotic signaling pathways. Relative expression of pro-apoptotic p53 target genes in microarray analysis of islets after 12 weeks of diet treatment. (n=4 per group)
